# Prevalence and Genetic Diversity of *Trueperella pyogenes* Isolated from Infections in European Bison (*Bison bonasus*)

**DOI:** 10.3390/ani12141825

**Published:** 2022-07-18

**Authors:** Ewelina Kwiecień, Ilona Stefańska, Magdalena Kizerwetter-Świda, Dorota Chrobak-Chmiel, Anna Didkowska, Wojciech Bielecki, Wanda Olech, Krzysztof Anusz, Magdalena Rzewuska

**Affiliations:** 1 Department of Preclinical Sciences, Institute of Veterinary Medicine, Warsaw University of Life Sciences, Ciszewskiego 8, 02-786 Warsaw, Poland; ilona_stefanska@sggw.edu.pl (I.S.); magdalena_kizerwetter_swida@sggw.edu.pl (M.K.-Ś.); dorota_chrobak@sggw.edu.pl (D.C.-C.); 2 Department of Food Hygiene and Public Health Protection, Institute of Veterinary Medicine, Warsaw University of Life Sciences, Nowoursynowska 159, 02-776 Warsaw, Poland; anna_didkowska@sggw.edu.pl (A.D.); krzysztof_anusz@sggw.edu.pl (K.A.); 3 Department of Pathology and Veterinary Diagnostics, Institute of Veterinary Medicine, Warsaw University of Life Sciences, Nowoursynowska 166, 02-787 Warsaw, Poland; wojciech_bielecki@sggw.edu.pl; 4 Department of Animal Genetics and Conservation, Institute of Animal Sciences, Warsaw University of Life Sciences, Ciszewskiego 8, 02-786 Warsaw, Poland; wanda_olech@sggw.edu.pl

**Keywords:** epidemiology, European bison, genetic diversity, RAPD-PCR typing, *Trueperella pyogenes*, virulence factors

## Abstract

**Simple Summary:**

In the European bison, *Trueperella pyogenes* is associated with different suppurative infections of mainly the urogenital tract or with abscesses in various tissues. Our research showed that in the studied population of European bison in Poland, the prevalence of *T. pyogenes* infections is relatively high. The significant genetic diversity of isolated strains was observed. However, in a few cases, the same RAPD profile was obtained for strains isolated from individuals living in the same area. Our study indicated that different virulence factors are related to the pathogenicity of this bacterium. The results obtained in this study provide valuable data about the prevalence, pathogenicity and genetic diversity of *T. pyogenes* in the European bison. Thus, it improves the knowledge on treatment for the European bison’s health and proves the importance of continuous monitoring for the protection of these wild ruminants.

**Abstract:**

*Trueperella pyogenes* is a Gram-positive bacterium causing purulent infections in many animal species, including the European bison. However, the data about the virulence and genetic relationships of *T. pyogenes* strains isolated from these wild ruminants are strongly limited. The aim of this study was to investigate the prevalence of *T. pyogenes* infections in the European bison, and to evaluate the genetic diversity of isolates from these animals. In the time span of 10 years, 328 European bison from 16 different locations were examined. The standard bacteriological methods were used for *T. pyogenes* isolation and identification from clinical specimens obtained from urogenital tract infections and abscesses of different locations. The presence of genes encoding known virulence factors was investigated by PCR, and the genetic diversity of *T. pyogenes* strains was examined with the RAPD-PCR method. The prevalence of *T. pyogenes* infections was 14.6%, and the pathogen was isolated from both female (47.9% of isolates) and male (52.1% of isolates) European bison. It should be highlighted that a considerable number of strains were isolated from the prepuce and penis infections. Therefore, the role of *T. pyogenes* in the pathogenesis of *balanoposthitis* should be seriously perceived. A total of 39 *T. pyogenes* strains were subjected to genetic characterization. All studied strains carried the *plo* gene, while the *nanH* (25.6%), *nanP* (23.1%), *cbpA* (7.7%), *fimA* (97.4%), *fimC* (69.2%), *fimE* (92.3%) and *fimG* (15.4%) genes were present with a variable frequency among the tested strains. The virulence genotype *plo*/*fimA*/*fimC*/*fimE* was dominant. RAPD-PCR typing showed a high level of genetic diversity among European bison *T. pyogenes* strains, and a total of 31 different RAPD profiles were distinguished. In a few cases, the same RAPD profile was found in strains obtained from animals living in the same area. This study provided the first data about the prevalence and genetic relationships of *T. pyogenes* in the Polish population of European bison. However, further epidemiological investigations are needed to understand the routes of transmission and dissemination of the pathogen in these wild animals.

## 1. Introduction

Nowadays, the global population of European bison (*Bison bonasus*) is categorized as “Near Threatened” by the International Union for Conservation of Nature (IUCN) Red List of Threatened Species. However, it is of importance that the population of these wild ruminants is constantly growing [1]. It should be mentioned that more than a quarter of the global European bison population lives in Poland. According to the European Bison Pedigree Book (EBPB) [2], the number of European bison in Poland at the end of 2021 was of 2429 individuals, including 206 in captivity and 2223 free living. The free-roaming European bison are grouped into eight herds of different sizes living in the Białowieska Primeval Forest, the Augustowska Forest, the Borecka Forest, the Knyszyńska Forest, the West Pomeranian, the Romincka Forest, the Janowskie Forest and the Bieszczady Mountains. However, the number of free-living herds of this species is planned to be systematically increased in the future. The captive herds are located in different regions of the country and generally consist of a low number of individuals [2].

Due to the fact that the current European bison population was derived from only a few founders, this population is characterized by low genetic variability [3]. Therefore, the animals are particularly susceptible to viral and bacterial infections, as well as to parasitic diseases. In addition, the increase in density also contributes to the spread of infectious diseases in herds. The health monitoring of the European bison population is a crucial element of species protection [4]. The available data about the health status of these mammals are quite limited. However, it is known that some of the viral infections or parasitic diseases are reported as a potential risk for the European bison’s health [1,5,6,7,8]. Moreover, various bacterial pathogens are related to infections in these wild ruminants. Recently, the bovine mycobacteria, as well as the atypical mycobacteria, were isolated from European bison kept in captivity in the area where bovine tuberculosis was diagnosed in the past [9,10]. In the serological survey of Q fever, antibodies against *Coxiella burnetii* were only found in a single free-living individual, suggesting that this disease is not currently a significant problem for the European bison’s health [11]. Other bacteria which may affect the European bison are *Treponema* spp., identified as a cause of digital dermatitis lesions [12]; *Pasteurella multocida*, associated with respiratory tract diseases [13] and *Borrelia burgdorferi* [14]. The Gram-positive bacteria from the genus *Trueperella*, including *Trueperella pyogenes*, *Trueperella bialowiezense* and *Trueperella bonasi*, were isolated from various infections in the European bison [15,16]. Two species, *T. bialowiezense* and *T. bonasi*, were obtained from the prepuce lesions in a European bison with *balanoposthitis* and were only reported in one study [15]. On the other hand, *T. pyogenes* was involved in different purulent infections in European bison and was isolated mainly from the respiratory tract and the urogenital tract of females and males [16]. The pathogenicity of this bacterium for the European bison is still poorly understood. Most of the available data regarding the characteristic of *T. pyogenes* strains are concentrated on isolates from livestock [17,18,19,20]. The known virulence factors of *T. pyogenes* include a pyolysin, the only known cytotoxin of this bacterium and a few adhesive factors, such as neuraminidases, fimbriae and extracellular matrix-binding proteins. Nevertheless, it seems that the known virulence factors of this bacterium occur with the variable frequency in strains regardless of their origin [19]. The evaluation of genetic diversity and relationships among bacterial strains is a very important aspect of epidemiological studies. So far, few molecular methods were applied for typing *T. pyogenes* strains isolated from various hosts [17,20,21,22,23,24,25]. However, the knowledge about the genetic diversity among *T. pyogenes* strains found in European bison is insufficient.

The aim of the present study was to investigate the prevalence of *T. pyogenes* isolated from infections among the European bison and to evaluate the genetic diversity of isolates to estimate their relationships. Moreover, the occurrence of selected virulence genes was studied. It should be mentioned that this research was conducted on unique clinical materials obtained from the endangered species, and for the first time the prevalence of *T. pyogenes* infections in the European bison was reported. As a result, the study provided valuable data about the European bison’s health and proved the importance of a continuous monitoring for its protection.

## 2. Materials and Methods

### 2.1. Material Collection

**Figure 1:**
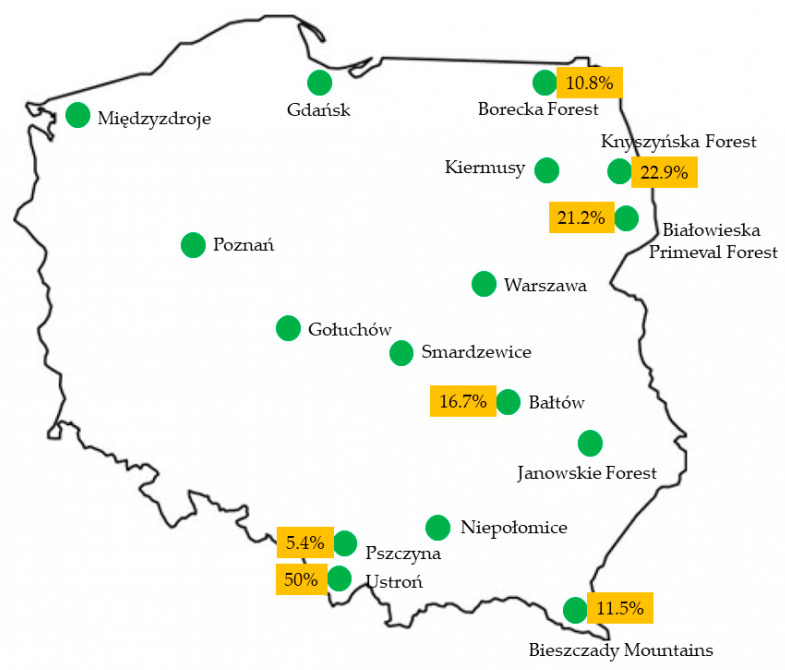
The location of herds of European bison in Poland (marked by green circles) from which the samples were collected. The prevalence of *T. pyogenes* infections in the studied areas was added in the yellow rectangles.

During the period from February 2011 to May 2021, different clinical specimens (*n* = 897), collected from 328 European bison, were subjected to a bacteriological examination at the Microbiological Diagnostic Laboratory, Department of Preclinical Sciences, Institute of Veterinary Medicine, Warsaw University of Life Sciences-SGGW in Poland. Animals originated from various regions of Poland (Figure 1) and the highest number of tested individuals was from the Białowieska Primeval Forest (*n* = 137), the Knyszyńska Forest (*n* = 35), the Borecka Forest (*n* = 37), Pszczyna (*n* = 37) and the Bieszczady Mountains (*n* = 26). Others, in lower numbers, were from Niepołomice (*n* = 12), Gołuchów (*n* = 11), Kiermusy (*n* = 7), Bałtów (*n* = 6), Warszawa (*n* = 5), Smardzewice (*n* = 4), Międzyzdroje (*n* = 3), Gdańsk (*n* = 3), Ustroń (*n* = 2), Poznań (*n* = 2) and Janów Lubelski (*n* = 1). Among the tested animals, 206 were free-living and 122 were kept in captivity. The specimens were taken from various purulent lesions of different locations from 94 animals. From 234 European bison, specimens were taken from animals without pathological changes, as a part of the health monitoring. Samples were collected from 215 live animals and from 113 dead animals, including 3 found dead of natural causes in the forest. The specimens were collected from 170 female and 152 male European bison, and in six cases, sex of animals was not noted. The majority of the tested animals were of unknown age; age information was only provided for 90 individuals (ranging from a few months to 22 years), including: ≤1 year; 30 animals, 2–10 years; 29 animals, ≥10 years; 31 animals). Swabs from mucous membranes or from abscesses were collected using sterile tubes with the Amies transport medium (Equimed, Kraków, Poland), the samples of internal organs were collected to sterile tubes (Equimed, Kraków, Poland) and all samples were stored at 4 °C and delivered to the laboratory under refrigerated conditions within 48 h.

The clinical materials were taken by qualified veterinarians during immobilization before transport of animals by placing a telemetric collar (these procedures did not require the consent of the ethics committee under Polish law) or were obtained as post mortem samples from animals who had died of natural causes or who were selectively culled for health reasons (the permission of General Directorate of Environment Protection in Warsaw number: WPN-I.6401.260.2015.EB.2, and the decision of The Polish Ministry of Environment, number: DOP-WPN.286.219.2018.MŚ). This study was a part of the project “Complex project of European bison conservation by State Forests” of the Forest Fund (Poland).

### 2.2. T. pyogenes Isolation and Identification

The specimens were cultured on Columbia Agar supplemented with 5% sheep blood (CAB) (Graso Biotech, Starogard Gdański, Poland) at 37 °C in 5% CO_2_ atmosphere for 48 h. Colonies displaying the growth features typical for *T. pyogenes* were selected for species identification. The cell morphology and the result of Gram staining, as well as a type of hemolysis on CAB, the catalase activity and the effect of the CAMP test with the *Staphylococcus aureus* ATCC 25923 reference strain, were evaluated. The preliminary isolates identification was confirmed by detection of the species-specific *plo* gene using PCR technique [16]. Pure cultures of *T. pyogenes* on CAB were suspended in the Tryptic Soy Broth (TSB) with 20% glycerol (*v*/*v*) and stored at −20 °C for future use. The single isolate was collected from each *T. pyogenes*-positive animal. However, in some cases, the pathogen was isolated from different locations in the same animal. In these cases, more isolates were collected from one animal. Only the stored *T. pyogenes* strains were subjected to further molecular investigations.

### 2.3. Genomic DNA Isolation

A genomic DNA was extracted from overnight cultures of the studied *T. pyogenes* strains in TSB, incubated at 37 °C in 5% CO_2_ atmosphere with agitation at approximately 100 rpm. The GeneJET^TM^ Genomic DNA Purification Kit (Thermo Fisher Scientific, Waltham, MA, USA) was used with minor modifications. Briefly, for each strain 1.5 mL of culture was centrifuged (10 min, 10000 rpm) and the pellet was suspended in 200 μL of lysis buffer containing 60 mg/mL lysozyme (Sigma-Aldrich, Steinheim am Albuch, Baden-Württemberg, Germany). The incubation at 37 °C with agitation for 30 min was performed. Subsequently, all the next steps were carried out according to the manufacturer’s instructions. The quantity and purity of the genomic DNA were determined with the spectrophotometer NanoDrop 1000 (Thermo Fisher Scientific, Waltham, MA, USA). The genomic DNA samples were stored at −20 °C until use.

### 2.4. Detection of Virulence Genes

The presence of eight genes encoding putative virulence determinants, such as pyolysin (Plo), neuraminidase H (NanH), neuraminidase P (NanP), collagen-binding protein (CbpA) and fimbrial subunits A, C, E, G (FimA, FimC, FimE, FimG) was investigated by PCR [17]. Sequences of the primer sets and reaction conditions are listed in Table 1. All amplification reactions were carried out in a 25 μL reaction mixture containing 8.5 μL of nuclease-free water (Thermo Fisher Scientific, Waltham, MA, USA), 12.5 μL of DreamTaq Green PCR Master Mix (Thermo Fisher Scientific, Waltham, MA, USA), 10 pmol of each primer (Genomed, Warsaw, Poland) and 70–80 ng of genomic DNA. Amplification products were recognized by electrophoresis (85 V by 45 min) in 1% (*w*/*v*) agarose gel in Tris-Acetate-EDTA (TAE) buffer with Midori Green DNA Stain (Nippon Genetics, Düren, Germany), visualized and analyzed using a Gel Doc^TM^ EZ Imaging System with Image Lab Software (version 5.2.1) (Bio-Rad, Hercules, CA, USA).

### 2.5. RAPD-PCR Typing of T. pyogenes Strains

The RAPD-PCR typing was performed with the M13 primer (5′-GAGGGTGGCGGTTCT-3′) (Eurofins Genomics, Ebersberg, Germany) according to our previous optimization study [25]. Each reaction mixture contained 12.5 µL of DreamTaq Green PCR Master Mix (Thermo Fisher Scientific, Waltham, MA, USA), 3.5 mM MgCl_2_ (Thermo Fisher Scientific, Waltham, MA, USA), 0.8 mM of each dNTP (Thermo Fisher Scientific, Waltham, MA, USA), 20 pmol of the M13 primer, 20 ng of the genomic DNA and nuclease-free water (Thermo Fisher Scientific, Waltham, MA, USA) up to a final volume of 25 µL. Thermal cycling conditions included initial denaturation at 94 °C for 3 min followed by 40 cycles of DNA denaturation at 94 °C for 30 sec, annealing for 30 sec at 45 °C and extension at 72 °C for 1 min with a final extension step of 72 °C for 7 min. Amplification products were recognized by electrophoresis (90 mA for 2.5 h) in 2% (*w*/*v*) agarose gel in Tris-Borate-EDTA (TBE) buffer with Midori Green DNA Stain (Nippon Genetics, Düren, Germany), visualized and preliminary analyzed using a Gel Doc^TM^ EZ Imaging System with Image Lab Software (version 5.2.1) (Bio-Rad, Hercules, CA, USA). The GeneRuler^TM^ 1 kb DNA Ladder (Thermo Fisher Scientific, Waltham, MA, USA) was used for estimating the molecular size of obtained bands.

The BioNumerics software version 7.6 (Applied Maths, Sint-Martens-Latem, Belgium) was used for the RAPD-PCR result analysis. The cluster analysis was performed by Unweighted Pair Group Method with Arithmetic Mean (UPGMA) using Dice similarity coefficient with optimization and position tolerance set at 1%. Strains were clustered using an 85% homology cut-off, above which strains were considered to be closely related and assigned to the same cluster.

The numerical index of discrimination (D) was calculated to evaluate the discriminatory power of the used RAPD-PCR method for *T. pyogenes* typing [26].

Two reference strains, *T. pyogenes* ATCC 19411 and *T. pyogenes* ATCC 49698, were included in the study (LGC Standards, Łomianki, Poland).

### 2.6. Statistical Analysis

Data were presented as counts and percentages. The Wilson score method was used to calculate 95% confidence intervals (CI 95%) for percentages. The Fisher’s exact test was used to compare a significant relationship between the presence of virulence genes and sex of European bison, as well as types of infection from which *T. pyogenes* strains were isolated. Only those virulence genes, which were not present in all tested *T. pyogenes* strains, such as *nanH*, *nanP*, *cbpA*, *fimA*, *fimC*, *fimE*, *fimG*, were included in this analysis. Types of lesions were grouped into four categories, such as (1) *balanoposthitis*, (2) lesions in the respiratory system, (3) lesions in the reproductive system (one strain obtained from the vagina without clinical lesions was excluded from the analysis) and (4) others (including abscesses or skin lesions). The level of significance was set at 0.05 (α = 0.05). A statistical analysis was performed in TIBCO Statistica 13.3.0 (TIBCO Software Inc., Palo Alto, CA, USA).

## 3. Results

### 3.1. Prevalence of T. pyogenes Infections in European Bison

*T. pyogenes* was isolated from 48 of 328 tested European bison (14.6%; CI 95%: 11.2%, 18.9%) (Supplementary Table S1). In 25 cases (52.1%, CI 95%: 38.3%, 65.5%) the pathogen was found in males and in 23 cases (47.9%; CI 95%: 34.5%, 61.7%) in females. In all these cases, *T. pyogenes* was isolated as a single pathogen. All of the isolated strains showed cell morphology typical for *T. pyogenes* (small, white colonies with the β-hemolysis zone), and were Gram-positive irregular rods, negatively reacting in the catalase test. In the CAMP test, all isolates showed a synergistic effect of the enhanced hemolysis.

**Figure 2:**
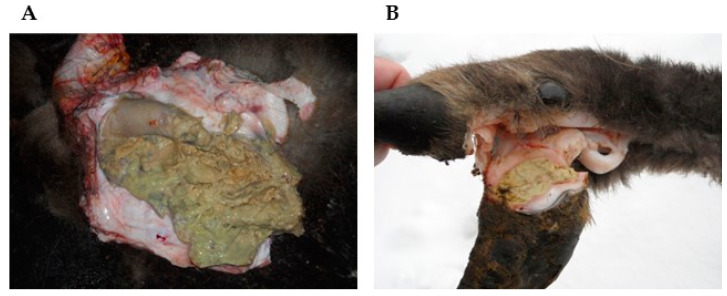
Examples of lesions related to *T. pyogenes* infections in European bison (*Bison bonasus*); (**A**): frequently occurred—*balanoposthitis* (the animal number 927), (**B**): rarely found—an interdigital abscess (the animal number 925).

Twenty of twenty-five (80%; CI 95%: 60.9%, 91.1%) *T. pyogenes* isolates from male European bison were obtained from the cases of balanoposthitis (Figure 2). In two of these bulls (8%; CI 95%: 2.2%, 25%), *T. pyogenes* was also found in the inguinal lymph node and in a nasal swab. In the remaining five male individuals, *T. pyogenes* was isolated from lung abscesses and purulent lesions on the neck, a forearm and a groin skin, as well as purulent lesions in the trachea or the lungs.

In female European bison, in 10 of 23 cases (43.5%; CI 95%: 25.6%, 63.2%), *T. pyogenes* was isolated from purulent lesions and discharges from the vagina. In 5 of 23 cases (21.7%; CI 95%: 9.7%, 41.9%), this bacterium was found in lung abscesses. In two of them (8.7%, CI 95%: 2.4%, 26.8%), *T. pyogenes* was also detected in a pericardial fluid and a nasal swab. In the remaining females, *T. pyogenes* was isolated from abscesses of the liver, the umbilical region, the submandibular gland and hoof lesions (Figure 2), as well as from a trachea and a nasal swab. In one case (4.3%; CI 95%: 0.8%, 21%), the pathogen (strain 11/2013) was obtained from the vagina without any clinical lesions.

*T. pyogenes* was isolated from the European bison coming from different regions (Figure 1), including the Białowieska Primeval Forest (*n* = 29), the Knyszyńska Forest (*n* = 8), the Borecka Forest (*n* = 4), Muczne in the Bieszczady Mountains (*n* = 3), Pszczyna (*n* = 2), Ustroń (*n* = 1) and Bałtów (*n* = 1).

In total, 52 *T. pyogenes* isolates were obtained from 48 animals. However, the isolates from 11 animals, including 7 isolates from *balanoposthitis* and 6 from other infections, were not stored, and were therefore excluded from further studies.

### 3.2. T. pyogenes Strains

A total of 39 *T. pyogenes* strains isolated from 37 European bison were stored and included in the molecular characterization study. These strains were not included in our previous studies [16,19], and therefore this is the first description of them. In the case of two pairs of strains, 26/2018 and 27/2018, as well as 33/2020 and 34/2020, they were isolated from the same individuals but from different clinical specimens.

The description of the studied strains is presented in Table 2.

### 3.3. Virulence Genes

All *T. pyogenes* strains (*n* = 39) carried the *plo* gene. The *nanH* and *nanP* genes were present in 10/39 (25.6%; CI 95%: 14.6%, 41.1%) and 9/39 (23.1%; CI 95%: 12.6%, 38.3%) strains, respectively. Among them, 6/39 (15.4%; CI 95%: 7.2%, 29.7%) strains carried both of these genes. The *cbpA* gene was present in 3/39 (7.7%; CI 95%: 2.7%, 20.3%) of the tested strains. The *fimA*, *fimC*, *fimE* and *fimG* genes were detected in 38/39 (97.4%; CI 95%: 86.8%, 99.5%), 27/39 (69.2%; CI 95%: 53.6%, 81.4%), 36/39 (92.3%; CI 95%: 79.7%, 97.3%) and 6/39 (15.4%; CI 95%: 7.2%, 29.7%) strains, respectively. All genes encoding tested fimbriae subunits were concurrently carried by 6/39 (15.4%; CI 95%: 7.2%, 29.7%) strains.

A statistically significant association between the type of infection and the presence of virulence genes was found in some cases (Table 3). The *nanH* gene was observed more frequently in *T. pyogenes* strains isolated from balanoposthitis (*p* = 0.0092), while the majority of strains isolated from the respiratory system lesions carried the *fimC* gene (*p* = 0.0445). Moreover, the *nanH* gene was not detected in *T. pyogenes* strains isolated from the lesions of female reproductive systems (*p* = 0.0424). Furthermore, the *nanH* gene was only noted in strains isolated from male European bison; however, it was not present in any strains isolated from females (*p* = 0.0001).

Eleven different virulence genotypes were identified. The genotype *plo/fimA/fimC/fimE*, detected in 12 strains, was dominant. In 15 strains, five or more virulence genes were present, in the following combinations: *plo/nanH/nanP/fimA/fimC/fimE* (six strains), *plo/cbpA/fimA/fimC/fimE/fimG* (three strains), *plo/nanH/fimA/fimC/fimE/fimG* (two strains), *plo/nanP/fimA/fimC/fimE/fimG* (one strain), *plo/nanP/fimA/fimC/fimE* (two strains), *plo/nanH/fimA/fimC/fimE* (one strain). In 12 strains, three or less virulence genes were carried, as follows: *plo/fimA/fmE* (nine strains), *plo/nanH/fimA* (one strain), *plo/fimA* (one strain) and *plo* (one strain). The data about virulence genotypes among tested *T. pyogenes* European bison strains are presented in Table 2.

### 3.4. RAPD Profiles and Relationships of T. pyogenes Strains

**Figure 3:**
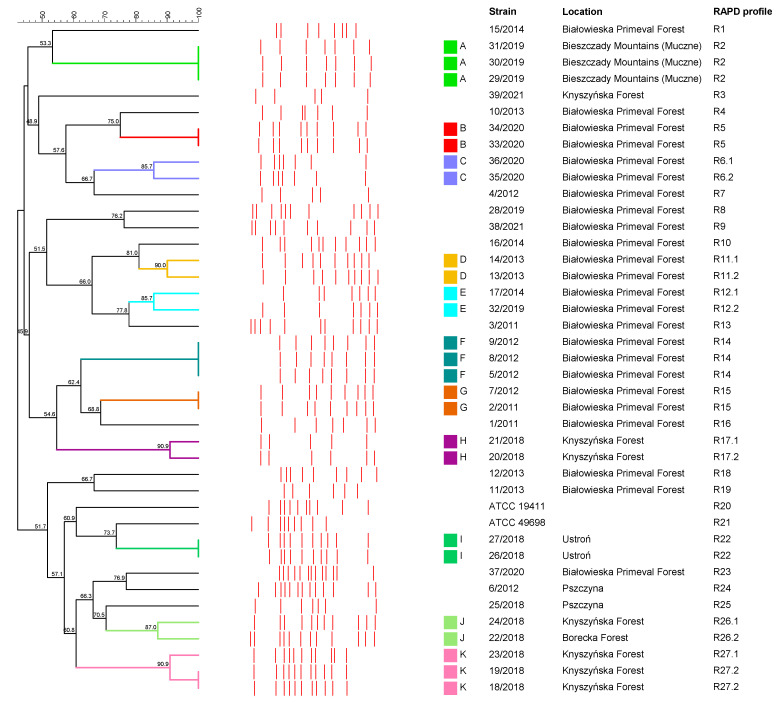
Dendrogram generated on the base of the results of RAPD-PCR typing of 39 *T. pyogenes* strains isolated from European bison and two *T. pyogenes* reference strains, using UPGMA analysis and Dice correlation coefficient. RAPD-PCR clusters (A–K) were based on the similarity cut-off of 85%.

The number of bands obtained in the RAPD-PCR typing with the M13 primer ranged from 5 to 13 (the average number of bands was 8.7) depending on the strain. Several very faint bands were excluded from the analysis. The obtained RAPD profiles included fragments in size from 463.66 bp to 2183 bp. Among the tested *T. pyogenes* strains isolated from European bison, 31 different RAPD profiles were distinguished. Both *T. pyogenes* reference strains included in this study also showed distinct RAPD profiles. Most of the *T. pyogenes* strains isolated from European bison (*n* = 25) presented unique RAPD profiles. Two RAPD profiles (R2 and R14) grouping three strains, as well as four RAPD profiles (R5, R15, R22 and R27.2) grouping two strains each, were extracted. The 33/2020 and 34/2020 strains with the R5 RAPD profile were isolated from the same European bison. Similarly, the 26/2018 and 27/2018 strains with the R22 RAPD profile were also obtained from one individual. All RAPD profiles defined for tested strains were described in Figure 3.

The dendrogram analysis, at 85% of the similarity, showed 11 clusters (designated from A to K) and 16 single strains (including two *T. pyogenes* reference strains) with unique RAPD-PCR profiles (Figure 3). The cluster A grouped three strains (29/2019, 30/2019 and 31/2019) obtained from three European bison living in the Bieszczady Mountains (Muczne) characterized by the same RAPD profile R2. Thirteen strains from the Białowieska Primeval Forest were reported within six clusters, such as B, C, D, E, F and G. The cluster B contained two strains (33/2020 and 34/2020) with the RAPD profile R5 isolated from one individual. The 5/2012, 8/2012 and 9/2012 strains with the RAPD profile R14 were classified to cluster F. In cluster G, 2/2011 and 7/2012 strains with the RAPD profile R15 were included. In clusters C, D and E strains were grouped with 85.7% (35/2020 and 36/2020), 90% (13/2013 and 14/2013) and 85.7% (17/2014 and 32/2019) of the similarity, respectively. Two clusters, H and K, included strains from the Knyszyńska Forest with 90.9% (20/2018 and 21/2018) and 90.9% (18/2018, 19/2018 and 23/2018) of the similarity, respectively. In cluster I, two strains with the RAPD profile R22 were located (26/2018 and 27/2018) and were obtained from the same individual from the Ustroń. The cluster J with 87% of the similarity included two strains, 22/2018 and 24/2018, which were isolated from the Borecka Forest and Knyszyńska Forest, respectively.

The RAPD-PCR typing of the European bison *T. pyogenes* strains in this study showed a relatively high value of the discrimination index, D = 0.987 (CI 95%: 0.982, 0.992).

## 4. Discussion

The conservation of the European bison requires a comprehensive approach based on several factors, including the setting of new herds, the management of environmental hazards and the monitoring of the population’s health status [27]. Due to the fact that the European bison is a wild and endangered species, clinical materials collected for any studies are unique and especially valuable. During the health monitoring, different specimens from European bison are taken, usually post mortem, for further diagnostics, microbiological, parasitological and other examinations. It is considered that the low genetic variability of this species may also have an impact on the health status of the individuals [3]. In Poland, two genetic lines of the species are distinguished in the European bison population, the Lowland (also known as Białowieża line) and the Lowland-Caucasian line. The European bison belonging to the Lowland genetic line is in the vast majority. The second largest, but unique, population of European bison from the Lowland-Caucasian genetic line lives in the Bieszczady Mountains [2,3]. Therefore, the continuous monitoring of the health of European bison populations is necessary, including genetic investigations.

The prevalence of *T. pyogenes* infections among European bison tested over the last 10 years estimated in this study was relatively high. For comparison, overall prevalence of *T. pyogenes* isolated from white-tailed deer (*Odocoileus virginianus*) in the USA was from 16.7% to 100%, depending on the location from the hunter-harvested [28]. Nevertheless, among 36 samples obtained from forest musk deer (*Moschus berezovskii*) kept in captivity in China, *T. pyogenes* was isolated from all of these samples [21]. In cattle, the prevalence of *T. pyogenes* among isolates from *mastitis* was 13.75% [24]. Moreover, this bacterium was often isolated as a main etiological agent of the infections. Based on the results of our bacteriological examinations, other opportunistic pathogens, such as *Corynebacterium* spp., *Streptococcus* spp., *P. multocida*, *Escherichia coli* and *Pseudomonas aeruginosa*, sporadically isolated from tested clinical materials (data not shown), may be involved in infections in European bison. Generally, *T. pyogenes* was mostly isolated from urogenital tract infections of European bison (62.5%; 30/48 *T. pyogenes*-positive animals), which is consistent with the findings in the previous study of *T. pyogenes* isolation in the years 2005–2010 [16]. It should be highlighted that in male European bison, in most cases, *T. pyogenes* in the pure culture was isolated from the prepuce and penis lesions. The same observation was reported in our previous work [16]. Thus, the role of this bacterium in the pathogenesis of *balanoposthitis* or *posthitis* seems to be relevant. Pathological changes in prepuce and penis characteristics for these diseases were found in 47.1% of the tested males in the Białowieska Primeval Forest between 2008 and 2013 [29]. However, *balanoposthitis* in European bison was already recognized as a serious health issue since 1980 [30]. This chronic disease may lead to reproduction problems, but despite the threat to the European bison population, the knowledge about the disease etiology is still poorly recognized.

In the female European bison in this study, the reproduction tract infections causing by *T. pyogenes* were noted mainly as suppurative lesions in the vagina or sporadically in the uterus, which is in accordance with previous findings [16,31] whereas, in cattle, *T. pyogenes* infections are mostly related to *metritis*, leading to impaired reproductive performance and milk production [17,18,24,32].

*T. pyogenes* causes infections in a broad range of host species, including wild animals, such as forest musk deer, white-tailed deer, fallow deer (*Dama dama*), roebuck (*Capreolus capreolus*), red deer (*Cervus elaphus*), bison (*Bison bison*) and camels [21,28,33,34,35,36]. This pathogen is often described as a cause of abscesses localized in various organs. Interestingly, in European bison, only in a few cases was this bacterium isolated from the liver abscesses; however, such lesions are common in cattle [37]. Moreover, in wild ruminants, *T. pyogenes* is not associated with mastitis, while such infections frequently occur in cattle. Nevertheless, regarding the pathogenic potential of *T. pyogenes*, it seems that this bacterium should be considered as a grave threat not only for livestock but also for wild animals such as the European bison. The usage of common pastures by cattle and European bison poses the risk of the transmission of bacteria among both species. The usage of agricultural lands by free-living European bison depends on the population management in the area. It was documented that the lack of feeding in a forest complex may lead to damages of field crops by European bison [38]. Furthermore, in some locations, the presence of European bison on fields is related to the population growth, as was the habit of foraging on undeveloped areas in the past. Moreover, the higher abundance of European bison population may often cause usage of forests and crops by them, which may lead to considerable damage in these areas [38]. Nevertheless, the available data concerning the transmission of *T. pyogenes*, as well as the dissemination of infections caused by this pathogen in wild animals are still strongly limited. Weather conditions and the density of the animal population were listed as factors associated with the frequency of *T. pyogenes* infections in free-living fallow deer [33]. Interestingly, our preliminary study showed that *Dermacentor reticulatus* ticks may be the possible vector of the *T. pyogenes* infections in European bison [39]. This research should be continued to confirm this route of pathogen transmission. On the other hand, due to the frequent isolation of *T. pyogenes* from lesions in European bison, the antimicrobial resistance of the obtained isolates is also worth consideration. Despite the fact that European bison are less exposed to the antibiotic pressure, it should be mentioned that our previous studies showed the resistance to tetracycline and kanamycin among *T. pyogenes* strains isolated from these animals [40,41]. Therefore, *T. pyogenes* occurring in European bison may be a potential reservoir of resistance determinants, including the tetracycline resistance genes detected previously [41]. The presence of antimicrobial resistant *T. pyogenes* strains in European bison could be a result of the direct or indirect contact with livestock, especially with cattle. However, this suggestion should be confirmed by the comparison of bovine and European bison *T. pyogenes* strains obtained from the same area.

The recognition of pathogen virulence and the detection of genes encoding virulence factors are essential for better understanding the pathogenesis of infectious diseases. Several virulence factors related to the pathogenicity of *T. pyogenes* are known [36]. The pyolysin, Plo, which leads to the lysis of the host cells, is regarded as the major one. The *plo* gene is detected in all *T. pyogenes* strains as the species-specific gene. Due to this, not surprisingly, the presence of this gene was demonstrated in all the *T. pyogenes* strains obtained from European bison in this study, as well as in previous research [16]. Among other virulence factors of *T. pyogenes*, the CbpA and different fimbriae subunits are involved in the adhesion to host cells. Moreover, two neuraminidases, NanH and NanP, probably contribute to the colonization of host tissues [36]. The results obtained in this study demonstrate lower percentage (7.7%, 25.6% and 23.1%, respectively) in the occurrence of *cbpA*, *nanH* and *nanP* in the European bison *T. pyogenes* strains compared to the previously reported research. In that study, the percentage of occurrence of *cbpA*, *nanH* and *nanP* was 12%, 40% and 44%, respectively [16]. In contrast, these genes are often found in the vast majority of bovine *T. pyogenes* strains [17,24,42,43]. However, in some studies, they were detected in a lower number of *T. pyogenes* isolates from cattle [18]. Nevertheless, it should be highlighted that the statistical analysis of the results showed a relationship between the isolation of *T. pyogenes* strains from lesions of the reproductive system, including *balanoposthitis* and the presence of *nanH*. High percentages of the European bison *T. pyogenes* strains in this study harbored the *fimA*, *fimC* and *fimE* genes, while the presence of the *fimG* gene was the lowest (15.4%). These results are consistent with the previous data [16]. The comparable results, the highest presence of *fimA*, *fimC* and *fimE*, and lower of *fimG*, were also noted for *T. pyogenes* strains isolated from cattle in Poland [18]. Generally, a high frequency of fimbrial genes was detected in the bovine strains in most studies [17,24,42,43], as well as among strains isolated from white-tailed deer [44,45]. Nevertheless, it seems that fimbriae subunits A and E are involved in the pathogenicity of *T. pyogenes* strains causing infections in European bison. The presence of virulence genotypes associated with five or more virulence genes in the European bison *T. pyogenes* strains suggested the importance of identification of these genes in order to determine the pathogenicity of this bacterium for these wild ruminants. Interestingly, one strain collected from the vagina without any pathological changes observed carried only *plo*. This suggests that strains isolated from healthy animals may be less virulent. However, this observation should be supported by the study of a larger number of strains isolated from clinically healthy European bison.

In this study, the RAPD-PCR method with the M13 primer was successfully used for the typing of *T. pyogenes* strains obtained from European bison from different locations in Poland. It should be highlighted that the method applied in this study was previously designed and optimized for the investigation of the genetic diversity of *T. pyogenes* strains of different origins, including isolates from European bison (however, those isolates were other than tested in this research) [25]. This typing method proved to be suitable for assessment of genetic diversity among *T. pyogenes* strains collected from European bison who inhabited various regions. The obtained results enabled the first detailed description of the relationship between *T. pyogenes* strains involved in infections in these wild ruminants. The index of discriminatory power obtained for typing of the European bison *T. pyogenes* strains in this study was higher than 0.90, and it is considered to be relevant for the reliable interpretation of discrimination levels of the RAPD-PCR typing [26].

The largest European bison population in Poland lives in the Białowieska Primeval Forest. Most of them are free-living individuals in a relatively large area. Thus, not surprisingly, high genetic diversity was observed among the strains (*n* = 24) isolated from these animals, since they were classified to twenty RAPD profiles. The most closely related strains (*n* = 7) from this region were grouped in three clusters represented by the same three RAPD profiles. However, three other clusters included the strains belonging to different RAPD profiles (from 85.7% to 90% of similarity). Nevertheless, eleven strains were characterized by unique RAPD profiles. As it was expected, the same RAPD profile of *T. pyogenes* was found in two cases of strains isolated from the same individual but from different clinical materials. All other strains, including those with the same RAPD profile, were obtained from various individuals. Despite the fact that the Białowieska Primeval Forest and the Knyszyńska Forest are adjacent to each other, and the fact that animals may move between both areas, the *T. pyogenes* strains obtained from the European bison living in these two locations were not closely related (from 48.9% to 60.8% of similarity). In the Knyszyńska Forest, the *T. pyogenes* strains (*n* = 7) associated with different infections were divided into two clusters, consisting of two and three strains, respectively (from 90.9% to 100% of similarity). Interestingly, one of the two remaining *T. pyogenes* strains from the Knyszyńska Forest showed a high similarity (87%) to the isolate from the Borecka Forest, and both strains were clustered together. However, this result can be explained by the near distance between these two forests. Importantly, a high relationship was detected among three *T. pyogenes* strains isolated from cases of *balanoposthitis* in three young bulls living in the enclosure in Muczne, Bieszczady Mountains. This finding indicates that the spread of *T. pyogenes* strains among European bison kept in captivity may be a serious threat for their health. Moreover, it confirms the possibility of dissemination of the infection caused by this pathogen in the European bison populations.

The prevalence and typing results obtained in the present study provided valuable epidemiological data to determine the significance of *T. pyogenes* infections in Polish populations of European bison. Based on our findings, this bacterium should be considered as one of the important etiological agents of infections in European bison, which may be transmitted between animals. Due to the status of the European bison as an endangered species, it is essential to further analyze the possible impact of *T. pyogenes* infections on the health of these animals. The subsequent epidemiological studies are required to elucidate the transmission routes for the monitoring and management of *T. pyogenes* infections in the European bison populations. This is especially important in light of an increasing density of free-living European bison herds that may favor the transmission of different infectious agents.

## 5. Conclusions

The prevalence of *T. pyogenes* infections in the tested population of European bison is worrying. The pathogen was often isolated from the studied clinical materials as a main etiological agent of purulent lesions. Due to this, the significant role of this bacterium in the etiology of *balanoposthitis* and other infections in the European bison should be seriously considered and must be monitored. The pathogenicity of the strains isolated from these wild ruminants is related to different virulence factors. Nevertheless, some of them appear to be more relevant in the virulence of the European bison *T. pyogenes* strains. The genetic diversity analysis showed that the strains isolated from European bison originated from the same locations may have the same, but sometimes also variable, RAPD profiles. Additionally it should be highlighted that the *T. pyogenes* strains of the same RAPD profile may occur in the European bison from the same area, suggesting a possible transmission of the pathogen among animals.

## Figures and Tables

**Table 1:** The primer sets and reaction conditions used in this study for PCR detection of virulence genes [17].

Target Gene	Primer Sequence (5′–3′)	Annealing Temperature (°C) *	Amplicon Size (bp)
*plo*	F–TCATCAACAATCCCACGAAGAG R–TTGCCTCCAGTTGACGCTTT	60	150
*nanH*	F–CGCTAGTGCTGTAGCGTTGTTAAGT R–CCGAGGAGTTTTGACTGACTTTGT	60	781
*nanP*	F–TTGAGCGTACGCAGCTCTTC R–CCACGAAATCGGCCTTATTG	60	150
*cbpA*	F–GCAGGGTTGGTGAAAGAGTTTACT R–GCTTGATATAACCTTCAGAATTTGCA	60	124
*fimA*	F–CACTACGCTCACCATTCACAAG R–GCTGTAATCCGCTTTGTCTGTG	57	605
*fimC*	F–TGTCGAAGGTGACGTTCTTCG R–CAAGGTCACCGAGACTGCTGG	60	843
*fimE*	F–GCCCAGGACCGAGAGCGAGGGC R–GCCTTCACAAATAACAGCAACC	55	775
*fimG*	F–ACGCTTCAGAAGGTCACCAGG R–ATCTTGATCTGCCCCCATGCG	57	929

* The thermal cycling conditions included initial denaturation at 94 °C for 3 min followed by 35 cycles of DNA denaturation at 94 °C for 1 min, annealing for 1 min at variable temperatures, and extension at 72 °C for 3 min with a final extension step of 72 °C for 7 min.

**Table 2:** Origin and virulence genotypes of the studied *T. pyogenes* strains (*n* = 39) isolated from European bison.

Strain Designation	Sex of Bison	Location	Type of Infection	Virulence Genotype
1/2011	Male	Białowieska Primeval Forest	*balanoposthitis*	*plo*/*fimA*/*fimE*
2/2011	Female	Białowieska Primeval Forest	abscess of umbilical region	*plo*/*nanP*/*fimA*/*fimC*/*fimE*
3/2011	Male	Białowieska Primeval Forest	*balanoposthitis*	*plo*/*fimA*/*fimC*/*fimE*
4/2012	Male	Białowieska Primeval Forest	abscesses of neck and forearm	*plo*/*nanH*/*fimA*
5/2012	Male	Białowieska Primeval Forest	purulent lesions of trachea	*plo*/*cbpA*/*fimA*/*fimC*/*fimE*/*fimG*
6/2012	Male	Pszczyna	purulent lesions of lungs	*plo*/*nanH*/*nanP*/*fimA*/*fimC*/*fimE*
7/2012	Female	Białowieska Primeval Forest	purulent lesions of trachea	*plo*/*fimA*/*fimE*
8/2012	Male	Białowieska Primeval Forest	purulent lesions of lungs	*plo*/*cbpA*/*fimA*/*fimC*/*fimE*/*fimG*
9/2012	Female	Białowieska Primeval Forest	purulent lesions of vagina	*plo*/*cbpA*/*fimA*/*fimC*/*fimE*/*fimG*
10/2013	Male	Białowieska Primeval Forest	*balanoposthitis*	*plo*/*nanH*/*fimA*/*fimC*/*fimE*/*fimG*
11/2013	Female	Białowieska Primeval Forest	vagina without clinical lesions	*plo*
12/2013	Female	Białowieska Primeval Forest	abscess of hoof	*plo*/*fimA*
13/2013	Male	Białowieska Primeval Forest	*balanoposthitis*	*plo*/*fimA*/*fimE*
14/2013	Male	Białowieska Primeval Forest	*balanoposthitis*	*plo*/*fimA*/*fimE*
15/2014	Male	Białowieska Primeval Forest	purulent lesions of groin skin	*plo*/*fimA*/*fimE*
16/2014	Male	Białowieska Primeval Forest	*balanoposthitis*	*plo*/*nanH*/*fimA*/*fimC*/*fimE*/*fimG*
17/2014	Male	Białowieska Primeval Forest	*balanoposthitis*	*plo*/*fimA*/*fimC*/*fimE*
18/2018	Female	Knyszyńska Forest	purulent discharge from uterus	*plo*/*fimA*/*fimE*
19/2018	Female	Knyszyńska Forest	purulent discharge from vagina	*plo*/*fimA*/*fimE*
20/2018	Female	Knyszyńska Forest	discharge from vagina	*plo*/*fimA*/*fimE*
21/2018	Female	Knyszyńska Forest	discharge from nose	*plo*/*fimA*/*fimC*/*fimE*
22/2018	Female	Borecka Forest	purulent lesions of vagina	*plo*/*fimA*/*fimE*
23/2018	Female	Knyszyńska Forest	purulent lesions of vagina	*plo*/*fimA*/*fimC*/*fimE*
24/2018	Male	Knyszyńska Forest	*balanoposthitis*	*plo*/*nanH*/*fimA*/*fimC*/*fimE*
25/2018	Female	Pszczyna	purulent lesions of vagina	*plo*/*nanP*/*fimA*/*fimC*/*fimE*/*fimG*
26/2018 *	Male	Ustroń	discharge from nose	*plo*/*nanH*/*nanP*/*fimA*/*fimC*/*fimE*
27/2018 *	Male	Ustroń	*balanoposthitis*	*plo*/*nanH*/*nanP*/*fimA*/*fimC*/*fimE*
28/2019	Male	Białowieska Primeval Forest	*balanoposthitis*	*plo*/*fimA*/*fimC*/*fimE*
29/2019	Male	Bieszczady Mountains (Muczne)	*balanoposthitis*	*plo*/*nanH*/*nanP*/*fimA*/*fimC*/*fimE*
30/2019	Male	Bieszczady Mountains (Muczne)	*balanoposthitis*	*plo*/*nanH*/*nanP*/*fimA*/*fimC*/*fimE*
31/2019	Male	Bieszczady Mountains (Muczne)	*balanoposthitis*	*plo*/*nanH*/*nanP*/*fimA*/*fimC*/*fimE*
32/2019	Female	Białowieska Primeval Forest	lung abscess	*plo*/*fimA*/*fimC*/*fimE*
33/2020 **	Female	Białowieska Primeval Forest	discharge from nose	*plo*/*fimA*/*fimC*/*fimE*
34/2020 **	Female	Białowieska Primeval Forest	lung abscess	*plo*/*fimA*/*fimC*/*fimE*
35/2020	Female	Białowieska Primeval Forest	purulent lesions of vagina	*plo*/*fimA*/*fimC*/*fimE*
36/2020	Female	Białowieska Primeval Forest	discharge from nose	*plo*/*fimA*/*fimC*/*fimE*
37/2020	Female	Białowieska Primeval Forest	purulent lesions of vagina	*plo*/*fimA*/*fimC*/*fimE*
38/2021	Female	Białowieska Primeval Forest	lung abscess	*plo*/*fimA*/*fimC*/*fimE*
39/2021	Female	Knyszyńska Forest	lung abscess	*plo*/*nanP*/*fimA*/*fimC*/*fimE*

* strains 26/2018 and 27/2018 were isolated from the same individual but from different clinical specimens. ** strains 33/2020 and 34/2020 were isolated from the same individual but from different clinical specimens.

**Table 3:** The prevalence of virulence genes in *T. pyogenes* strains isolated from different types of infections in European bison (*n* = 38).

Virulence Gene	*n* (%) of *T. pyogenes* Strains Isolated From *			
	*Balanoposthitis*	Lesions in the Respiratory System	Lesions in the Reproductive System in Cow	Other Lesions
*plo*	13 (100)	12 (100)	9 (100)	4 (100)
*nanH*	7 (53.8) **	2 (16.7)	0 (0) ***	1 (25)
*nanP*	4 (30.8)	3 (25)	1 (9)	1 (25)
*cbpA*	0 (0)	2 (16.7)	1 (9)	0 (0)
*fimA*	13 (100)	12 (100)	9 (100)	4 (100)
*fimC*	10 (76.9)	11 (91.7) ****	5 (55.6)	1 (25)
*fimE*	13 (100)	12 (100)	9 (100)	2 (50)
*fimG*	2 (15.4)	2 (16.7)	2 (22.2)	0 (0)

* one strain (11/2013) obtained from the vagina without clinical lesions was excluded from the analysis. ** *p* = 0.0092; *** *p* = 0.0424; **** *p* = 0.0445.

## Data Availability

Raw data are available upon request from the corresponding authors.

## Supplementary Materials

The following supporting information can be downloaded at: https://www.mdpi.com/article/10.3390/ani12141825/s1, Supplementary Table S1. The detailed characterization of European bison (*Bison bonasus*) from which *Trueperella pyogenes* was isolated in this study.
