# Supplementary material for: Prevalence and Genetic Diversity of Trueperella pyogenes Isolated from Infections in European Bison (Bison bonasus)
Source: Animals (Basel). 2022 Jul 18;12(14):1825. doi: 10.3390/ani12141825 (PMC9311551; doi:10.3390/ani12141825)
Supplement: Supplementary file 1 [file animals-12-01825-s001.zip › animals-1774061-supplementary.pdf]

**Table S1.** The detailed characterization of European bison (*Bison bonasus*) from which *Trueperella pyogenes* was isolated in this study.

| Year of studying | Animal designation | Sex | Age | Location* | Type of herd | Condition | Collected samples | *Trueperella pyogenes* isolate (from) |
|---|---|---|---|---|---|---|---|---|
| **2011** | L555 | M | 18 years | BPF | free-living | dead** | foreskin swab | 1/2011 (foreskin swab) |
| | 893/F | F | 5.5 years | BPF | free-living | dead | lung swab, vaginal swab, skin abscess swab, lymph node abscess swab | 2/2011 (skin abscess swab) |
| | 1/BPF | M | unknown | BPF | free-living | dead | foreskin swab | 3/2011 (foreskin swab) |
| **2012** | 2/BPF | M | unknown | BPF | free-living | alive | skin abscess swab | 4/2012 (skin abscess swab) |
| | 908 | F | unknown | BPF | free-living | dead | salivary gland abscess swab | 1_nt*** (salivary gland abscess swab) |
| | 914 | M | 6 years | BPF | free-living | dead | foreskin swab, tracheal abscess swab | 5/2012 (tracheal abscess swab) |
| | „Plesar" | M | unknown | P | captive | dead | internal organs, lung abscess swab | 6/2012 (lung abscess swab) |
| | 918 | M | 2.5 years | BPF | free-living | dead | foreskin swab | 2_nt (foreskin swab) |
| | 919 | F | 22 years | BPF | free-living | dead | lung swab, vaginal swab, tracheal swab | 7/2012 (tracheal swab) |
| | 920 | M | 4-5 months | BPF | free-living | dead | foreskin swab, lung swab | 8/2012 (lung swab) |
| | 921 | F | unknown | BPF | free-living | dead | vaginal swab, lung swab | 9/2012 (vaginal swab) |
| **2013** | 922 | M | 5 years | BPF | free-living | dead | foreskin swab, tracheal swab, urethral swab | 10/2013 (foreskin swab) |
| | 923 | F | 6 years | BPF | free-living | dead | vaginal swab, cervical swab | 11/2013 (vaginal swab) |
| | 925 | F | 5-6 months | BPF | free-living | dead | vaginal swab, hoof abscess swab | 12/2013 (hoof abscess swab) |
| | 926 | M | 3 years | BPF | free-living | dead | foreskin swab, lymph node | 13/2013 (foreskin swab) |
| | 927 | M | 2.5 years | BPF | free-living | dead | foreskin swab | 14/2013 (foreskin swab) |
| | 932 | M | unknown | BPF | free-living | dead | foreskin swab, lung swab | 3_nt (foreskin swab) |
| | 935 | M | unknown | BPF | free-living | dead | foreskin swab | 4_nt (foreskin swab) |
| **2014** | 1/BF | M | unknown | BF | free-living | dead | foreskin swab, lymph node, gut | 5_nt (foreskin swab), 6_nt (lymph node) |
| | 942/M | M | unknown | BPF | free-living | dead | foreskin swab, skin swab, skin slice, uterine contents | 15/2014 (skin swab) |
| | 944/M | M | unknown | BPF | free-living | dead | foreskin swab | 16/2014 (foreskin swab) |

| | | | | | | | | |
|---|---|---|---|---|---|---|---|---|
| | 945/M | M | unknown | BPF | free-living | dead | foreskin swab | 17/2014 (foreskin swab) |
| | 947/F | F | unknown | BPF | free-living | dead | liver, labia minora swab | 7_nt (liver) |
| **2018** | 1/KF | F | unknown | KF | free-living | dead | uterus pus | 18/2018 (uterus pus) |
| | 2/KF | F | 2.5-3 years | KF | free-living | alive | nasal swab, conjunctival swab, pus from a vagina | 19/2018 (vaginal pus) |
| | L-1302 | M | unknown | KF | free-living | alive | nasal swab, conjunctival swab, foreskin swab | 8_nt (foreskin swab) |
| | L-1304 | F | unknown | KF | free-living | alive | nasal swab, conjunctival swab, vaginal swab, vaginal mucus | 20/2018 (vaginal mucus) |
| | L-1320 | F | unknown | KF | free-living | alive | nasal swab, conjunctival swab, vaginal swab | 21/2018 (nasal swab) |
| | L-1323 | F | unknown | BF | free-living | alive | nasal swab, conjunctival swab, vaginal swab | 22/2018 (vaginal swab) |
| | L-1324 | F | unknown | BF | free-living | alive | nasal swab, conjunctival swab, vaginal swab | 9_nt (vaginal mucus) |
| | L-986 | M | unknown | BF | free-living | alive | nasal swab, conjunctival swab, foreskin swab | 10_nt (foreskin swab) |
| | L-1331 | F | unknown | KF | free-living | alive | nasal swab, conjunctival swab, vaginal swab | 23/2018 (vaginal swab) |
| | L-1339 | M | unknown | KF | free-living | alive | nasal swab conjunctival swab, foreskin swab | 24/2018 (foreskin swab) |
| | L-1347 | M | unknown | B | captive | alive | nasal swab, conjunctival swab, foreskin swab | 11_nt (foreskin swab) |
| | L-1358 | F | unknown | P | captive | alive | nasal swab, conjunctival swab, vaginal swab | 25/2018 (vaginal swab) |
| | L-1372 | M | unknown | U | captive | alive | nasal swab, conjunctival swab, foreskin swab | 26/2018 (nasal swab), 27/2018 (foreskin swab) |
| **2019** | L-1536 | F | 15 years | BPF | free-living | dead | nasal swab, conjunctival swab, vaginal swab, lung pus, pleural fluid, pericardial fluid | 12_nt (pericardial fluid), 13_nt (lung pus) |
| | L-1556 | M | 12 years | BPF | free-living | dead | nasal swab, conjunctival swab, pleural fluid, foreskin swab | 28/2019 (foreskin swab) |
| | KB792 | M | 1 year | BM | captive | alive | nasal swab, conjunctival swab, foreskin swab | 29/2019 (foreskin swab) |

| | | | | | | | | |
|---|---|---|---|---|---|---|---|---|
| | KB796 | M | 1 year | BM | captive | alive | nasal swab, conjunctival swab, foreskin swab | 30/2019 (foreskin swab) |
| | KB797 | M | 1 year | BM | captive | alive | nasal swab, conjunctival swab, foreskin swab | 31/2019 (foreskin swab) |
| | L-1570 | F | 19 years | BPF | free-living | dead | nasal swab, conjunctival swab, vaginal swab, lung pus, tracheal abscess swab | 32/2019 (lung pus) |
| **2020** | L-1575 | F | 17 years | BPF | free-living | dead | nasal swab, conjunctival swab, vaginal swab, lung pus, pericardial fluid | 33/2020 (nasal swab), 34/2020 (lung pus) |
| | L-1668 | F | unknown | BPF | free-living | alive | nasal swab, conjunctival swab, vaginal swab | 35/2020 (vaginal swab) |
| | L-1669 | F | unknown | BPF | free-living | alive | nasal swab, conjunctival swab, vaginal swab | 36/2020 (nasal swab) |
| | L-1672 | F | unknown | BPF | free-living | alive | nasal swab, conjunctival swab, vaginal swab | 37/2020 (vaginal swab) |
| **2021** | 18-201 | F | unknown | BPF | free-living | dead | internal organs, lung pus | 38/2021 (lung pus) |
| | L-1758 | F | 8 years | KF | free-living | dead | lung pus | 39/2021 (lung pus) |

*BPF – Białowieska Primeval Forest, KF – Knyszyńska Forest, BF – Borecka Forest, P – Pszczyna, U – Ustroń, B – Bałtów, BM – Bieszczady Mountains

** - „dead" – selectively culled for health reasons

*** nt - an isolate not stored and not tested in the study
